# Characteristics of care methods for daily life disabilities in Alzheimer's type dementia that respect autonomy and independence

**DOI:** 10.1002/nop2.283

**Published:** 2019-04-15

**Authors:** Sayuri Suwa, Akiyo Yumoto, Mari Ueno, Tomoko Yamabe, Yumiko Hoshishiba, Mihoko Sato

**Affiliations:** ^1^ Graduate School of Nursing Chiba University Chiba Japan; ^2^ Graduate School of Nursing Jichi Medical University Tochigi Japan; ^3^ Japan Visiting Nursing Foundation Tokyo Japan; ^4^ Mitsubishi UFJ Research & Consulting Co., Ltd. Tokyo Japan

**Keywords:** Alzheimer's type dementia, autonomy, daily life disability care, independence

## Abstract

**Aim:**

To clarify the characteristics of appropriate care methods for people with daily life disabilities due to Alzheimer's type dementia.

**Design:**

A cross‐sectional survey study.

**Methods:**

A survey was implemented targeting 2,156 advanced care practitioners for dementia. The postal, self‐administered anonymous questionnaire was rated on a 4‐point Likert scale to assess the benefits of care for daily life disabilities depending on severity. We conducted factor analysis to determine characteristics of the appropriate care.

**Results:**

There were 568 valid responses, a valid response rate of 26.3%. The characteristics found were “Simplicity of necessities” and “Communication using verbal language on what should be done next” for mild cases; “Opportunities for completion of a task are provided with verbal communication,” “Marks” and “Arrange the environment with verbal communication” for moderate cases; and “Explain the process in the order of each individual action,” “Prevent non‐starts and interruptions” and “Confirm intention” for severe cases.

## 1 INTRODUCTION

The number of elderly people with dementia in Japan was estimated to be 4.62 million in 2012, with the prevalence of dementia among older people estimated to be around 15% (Ikejima et al., 2012; Ministry of Health, Labour, & Welfare, 2013). According to a nationwide survey conducted from April 2011–March 2013, Alzheimer's type dementia is the most common primary disease of dementia, accounting for 67.6% (Ministry of Health, Labour, & Welfare, 2013). A longitudinal study in the towns of Hisayama‐cho in Fukuoka Prefecture and Daisen‐cho in Tottori Prefecture has ascertained that the prevalence of Alzheimer's type dementia is increasing over time (Sekita et al., 2010; Wakutani et al., 2007).

Based on this situation in Japan, a community‐based integrated support and service provision system is expected to be constructed and operational by 2025, to enable elderly people to continue living in familiar communities and on their own terms until the end of their lives, whenever possible. This strategy is based on the aim of supporting independent living and retaining the dignity of older people (Ministry of Health, Labour, & Welfare, 2012). In 2015, the New Orange Plan was revealed, which includes seven policies regarding dementia such as the provision of timely and appropriate medical treatment and nursing care suited to the condition of the dementia; this policy was enacted in anticipation that the number of elderly people with dementia would reach approximately 7 million by 2025 (Ministry of Health, Labour, & Welfare, 2015).

### 1.1 Background

Dementia care in Japan is based on person‐centred care advocated by Kidwood (1997). The aim has been to improve the quality of dementia care through studies on cognitive impairment and behavioural and psychological symptoms of dementia (BPSD), which are predominantly characteristics of Alzheimer's type dementia and by developing nationwide public training on dementia medical treatment and nursing care (Ministry of Health, Labour, & Welfare, 2006,2016). However, previously in Japan, long‐term care facilities, day services and geriatric hospitals used by many people with dementia have referenced indices such as the Barthel Index (Mahoney & Barthel, 1965) regarding the activities of daily living (ADL) for people with dementia. These indices only employ a phased assessment, classifying the person as independent, or requiring partial care or total care, without specifying what each of these types of care entails. Thus, traditional methods of care have been employed by individual caregivers and care facilities. Methods for assessing ADL and instrumental activities of daily living (IADL) of older people, such as the Physical Self‐Maintenance Scale (Lawton & Brody, 1969) and Instrumental ADL Scale (Lawton & Brody, 1969), have been developed; however, these scales have not been fully used for planning care in Japan. A systematic review by Prizer and Zimmerman focused on dressing, toileting and meals among the ADL of people with dementia and clarified progressive support methods and associated evidence for these methods (Prizer & Zimmerman, 2018). However, this study did not clarify support methods and associated evidence for ADL other than dressing, toileting and meals.

Reports show that it is important to appropriately ascertain deficits in executive function (executive function disorders) in cognitive impairment, to support execution of daily tasks, including ADL, in the daily lives of people with dementia (Nakaaki & Sato, 2015). Execution function is a series of cognitive functions to facilitate decision‐making, planning, execution and modification, which are essential for humans to behave with purpose in the environment (Lezak, Howieson, Loring, Hannay, & Fischer, 2004; Walsh, 1991). However, disorders other than execution function disorders also make it difficult for people to execute various daily tasks by themselves, including attention disorders, agnosia and apraxia. Asada (2015) regards daily tasks that are difficult to execute due to the effect of dementia as daily life.

Several assessment scales have been developed to date for deficits in executive function (executive function disorders) in ADL in patients with Alzheimer's type dementia (Sato et al., 2011; Shido, Matsuda, & Saito, 2008; Skurla, Rogers, & Sunderland, 1988). However, the results of assessing people with dementia using these ratings are not used for care, and as mentioned above, currently assistance for ADL is implemented based on categorizing the person as independent or requiring partial care or total care. In addition, there has been no clarification of daily life disabilities caused by cognitive impairment, including execution function disorders, nor specific methods for providing care to support daily life disabilities.

The Diagnostic and Statistical Manual of Mental Disorders (DSM‐5) describes execution function and complex attention as different cognitive domains (American Psychiatric Association, 2013); however, in reality, maintenance, selection and distribution of attention are necessary conditions for establishing execution functions (Nakaaki & Sato, 2015). Oikawa, Oguri, Sato, and Imamura (2006) also claim that the cognitive functions of maintaining attention and distractibility, retaining information, recalling past information and determining time and location are required simultaneously when a person encounters a situation that requires executive function for ADL. Therefore, given that it is impossible to correlate a single cause of daily life disability with a single cognitive impairment, it is also impossible to gain an appropriate understanding of daily life disability and to provide support using assessment that focusses on individual execution function disorders in people with dementia.

Thus, it is vital to clarify the specifics of the kind of care that should be provided for daily life disability depending on specific cognitive impairment, including execution function disorders, to support the autonomy and independence of people with dementia, focusing on Alzheimer's type dementia, to enable people to live in familiar areas and on their own terms until the end of their life whenever possible.

### 1.2 Aim

The aim of this study was to clarify characteristics of the appropriate care for Alzheimer's type dementia patients' daily life disabilities. Such care on the part of advanced care practitioners in medical or care facilities or home care services (hereinafter referred to as advanced practitioners) must respect patients' autonomy and independence and incorporate advanced knowledge and techniques in dementia care in line with the severity of patients' Alzheimer's type dementia.

This study investigated the characteristics of dementia care provided by advanced care practitioners for persons with mild, moderate and severe cognitive impairment due to Alzheimer's type dementia when they are willing to undertake ADL.

In this study, advanced care practitioners for dementia were defined as nurse specialists and certified nurses, including dementia care leaders, certified nurse specialists in gerontological nursing and certified nurses in dementia nursing. The reason for this definition is firstly that training and education of dementia care leaders was started in 2000, to train them to plan, operate and guide dementia care practitioner training and dementia care practice leader training (Onodera, 2007; Suwa, 2010). Employing dementia care leaders is one of the requirements for determining whether additional specialized dementia care is necessary (Ministry of Health, Labour, & Welfare, 2009); thus, long‐term care fees for businesses implementing advanced dementia care practice are influenced by this factor. The Japanese Nursing Association has also started a nurse specialist system that fulfils the role of practice, consultation, coordination, ethics coordination, education and research and a certified nurse system that fulfils the role of practice, guidance and consultation (Japanese Nursing Association, 2016a,2016b). These gerontological certified nurse specialists and certified nurses in dementia nursing are now developing advanced dementia nursing activities.

### 1.3 Design

This study employs a cross‐sectional research design.

## 2 METHOD

### 2.1 Sample/Participants

In this study, we wanted to target professions highly specialized in dementia care, with experience in care for people with dementia living at home. Therefore, we targeted 2,156 nurses comprised of 62 certified nurse specialists in gerontological nursing, 22 certified nurse specialists in community health nursing, 11 certified nurse specialists in home care nursing, 363 certified nurses in visiting nursing and 409 certified nurses in dementia nursing whose names and affiliations were published on the Japanese Nursing Association website (accessed September 2014), as well as 1,289 dementia care leaders who completed training by FY2013 in two (Tokyo and Obu) of the three dementia care research and training centres established nationwide.

### 2.2 Data collection

#### 2.2.1 Survey implementation period

We implemented a postal self‐administered anonymous survey from November 2014–January 2015. During this period, one reminder letter was sent to all recipients urging them to respond to the survey.

#### 2.2.2 Development of the questionnaire

Care methods were selected for daily life disabilities involving 19 daily tasks (go to target location, defecation, urination, hand washing, face washing, teeth brushing, eating meals, drinking, dressing, wearing shoes, undressing, bathing, transfers, lying down, applying makeup, shaving, cleaning dentures, managing medication and expressing intent), which were regarded as effective for supporting autonomy and independence by the research team and were considered to serve as hints applicable to care for a variety of daily life disabilities. This information was selected from previous research on good practices of dementia care to support autonomy and independence in line with the daily life disabilities in Alzheimer's type dementia in people cared for at home or in long‐term care facilities, whose cognitive impairment is at functional assessment staging (FAST) stage 3 (mild), FAST stage 4 (moderate) or FAST stage 5–7 (severe) based on Functional Assessment Staging (Japan Visiting Nursing Foundation, 2003; Ministry of Health, Labour, & Welfare, 2013; Reisberg, 1988). Ultimately, 127 items were selected that expressed daily life disabilities and care methods in a single sentence, in line with the severity of the cognitive function disorder for the 19 daily tasks. The items were selected by an expert council who served as delegates for the Japanese Society for Dementia Care and who were experts in dementia care (nurses, care workers and occupational therapists). Furthermore, 17 items on daily life disabilities and associated care for nine daily tasks were set as questions on care for people with daily life disabilities in Alzheimer's type dementia and mild cognitive impairment. Similarly, 36 items on daily life disabilities and associated care for 18 daily tasks were set as questions on care for people with daily life disabilities in Alzheimer's type dementia and moderate cognitive impairment. Finally, 74 items on daily life disabilities and associated care for 18 daily tasks were set as questions on care for people with daily life disabilities in Alzheimer's type dementia and severe cognitive impairment.

We developed a self‐administered anonymous survey about the extent of benefits of care for each level of severity of cognitive impairment that enables autonomy and independence for people with daily life disabilities in Alzheimer's type dementia using a 4‐point Likert scale: 4 = Beneficial, 3 = Somewhat beneficial, 2 = Not very beneficial, 1 = Not beneficial. In this context, *beneficial care* is care that allows even patients with daily life disabilities due to Alzheimer's type dementia to proactively engage in daily activities of their own free will as much as possible.

#### 2.2.3 Validity and reliability/rigour

To increase the reliability, validity and rigour of the survey, first, using the developed survey form, we requested a pre‐test from two nurses, one occupational therapist and one certified care worker both of whom had been carrying out dementia care for 20 years or longer. The results revealed that there were no problems with the survey form. However, because it was essential that subjects accurately understood the severity of cognitive impairment due to Alzheimer's type dementia, FAST was enclosed with the questionnaire to enable the respondent to reference it while answering the questions. Additionally, the respondents were asked to respond to the questionnaire from the viewpoint of whether the care would be beneficial to many people with Alzheimer's type dementia for each category of severity, rather than based on the perspective of care for a particular person with Alzheimer's type dementia.

### 2.3 Analysis

The subject attributes were summarized with descriptive statistics. Exploratory factor analysis was implemented with the maximum likelihood method and promax rotation for each level of cognitive impairment severity using response data relating to the benefits of care for the 127 items of daily life disability. In this way, we hoped to clarify factors that comprise the benefits of care for daily life disability that respects the autonomy and independence of people with Alzheimer's type dementia.

### 2.4 Ethics

Implementation of the survey was approved after review by the research ethics committee of the Japan Visiting Nursing Foundation. Specifically, the aim of the research, strict protection of privacy and the voluntary nature of survey collaboration, was explained in writing and consent to participate in the survey was assumed by the respondent completing and returning the self‐administered anonymous survey.

## 3 RESULTS

### 3.1 Valid response rate

There were 568 valid responses from 2,156 advanced care practitioners for dementia, indicating a valid response rate of 26.3%.

### 3.2 Subject attributes

The gender of the respondents was 76.1% female and 23.9% male (Table 1). The largest age group was respondents in their 40s, comprising 41.2%. There were multiple answers for respondents' qualifications; nurses comprised the largest group, accounting for 61.4%, followed by long‐term care support specialists (44.9%) and care workers (33.8%). Qualifications as advanced care practitioners for dementia included dementia care leaders (45.8%), certified nurses in dementia nursing (30.8%), dementia care specialists (19.7%) and certified nurse in visiting nursing (16.9%) (from highest to lowest). The most common affiliated facility was visiting nursing stations (17.1%), followed by general hospital wards (16.0%), long‐term intensive care facilities for older people (12.5%) and group homes (12.1%). The most common range of number of years' experience in dementia care was between 10–15 years, accounting for 38.6%.

**Table 1:** Characteristics of participants (*N* = 568)

	*N*	%
Gender		
Male	136	23.9
Female	432	76.1
Age group		
20s	1	0.2
30s	134	23.6
40s	234	41.2
50s	143	25.2
60s	53	9.3
70s	3	0.5
Licence (Multiple answers)		
Nurse	349	61.4
Care manager	255	44.9
Care worker	192	33.8
Social worker	49	8.6
Home helper	35	6.2
Public health nurse	24	4.2
Midwife	5	0.9
Physical therapist	4	0.7
Occupational therapist	1	0.2
Speech therapist	0	0.0
Medical doctor	0	0.0
No answer	1	0.2
Others	36	6.3
Qualification (Multiple answers)		
Dementia care leader	260	45.8
Certified nurse in dementia nursing	175	30.8
Dementia carer qualified	112	19.7
Certified nurse in visiting nursing	96	16.9
Certified nurse specialist in gerontological nursing	19	3.3
Advanced dementia carer qualified	15	2.6
Certified nurse specialist in home care nursing	4	0.7
Certified nurse specialist in community health nursing	2	0.4
No answer	7	1.2
Others	14	2.5
Affiliation		
Home visiting nursing station	97	17.1
General hospital ward	91	16.0
Intensive care home for older people	71	12.5
Group home for people with dementia	69	12.1
Long‐term care health facility	42	7.4
Day service	27	4.8
Sanatorium medical facility for older people requiring long‐term care	26	4.6
Discharge support section in hospital	23	4.0
Long‐term care support office	18	3.0
Combined multiple service	17	3.0
Others	85	14.9
No answer	2	0.4
Years of dementia care experience		
Less than 10 years	104	18.3
10 years or more and <15 years	219	38.6
15 years or more and <20 years	124	21.8
Over 20 years	108	19.0
No answer	13	2.3

### 3.3 Characteristics of care that is beneficial

Factor analysis (promax rotation) was conducted to clarify factors that comprise the benefits of care common to various daily tasks using responses obtained with a 4‐point Likert scale to determine the degree of benefit of care for those items to facilitate autonomy and independence of the 127 items of daily life disabilities.

Two factors were extracted (Table 2) for the usefulness of items with a factor loading of 0.4 or more as the components of benefits relating to care for people with daily life disabilities in Alzheimer's type dementia and mild cognitive impairment. The first factor was named “Simplicity of necessities: integrate the necessities for daily living in a simplified format,” and the second factor was named “Communication using verbal language on what should be done next: speak to the person and inform them of what they should do next.” Cronbach's *α* coefficient demonstrated internal consistency (0.843, 0.774, for the first and second factors, respectively), while the cumulative contribution ratio was 40.99%.

**Table 2:** Benefits of care for daily living disabilities in people with Alzheimer's type dementia and mild cognitive impairment

Variable			Factor 1	Factor 2
Factor 1: Simplicity of necessities: integrate the necessities for daily living in a simplified format				
Cronbach's *α*	0.8433			
Enable the person to take their medication at the correct time using a medication calendar			0.8311	−0.1116
Implement measures to enable confirmation that the person took the medication, such as writing the date on the packaging			0.7295	−0.0527
Consult with the doctor to change the packaging to one dose packages, to enable the person to take their medication at the correct time			0.6793	−0.0092
Write notes and enable the person to take it with them when shopping			0.6719	−0.0055
Ensure the person eats food before the expiry date by writing notes and affixing them to the refrigerator			0.6187	0.0573
The caregiver is to inform the person of the garbage collection day			0.5091	0.2831
Consult with the doctor to enable the patient to take medication once a day, to enable the person to take their medication at the correct time			0.4225	0.0426
Ensure the caregiver communicates the time to take medication, to enable the person to take their medication at that time			0.4035	0.2843
Factor 2: Communication using verbal language on what should be done next: speak to the person and inform them of what they should do next				
Cronbach's *α*	0.7741			
Ensure the person knows how to hang clothes out by the caregiver standing alongside and communicating the procedures			−0.0492	0.7177
Enable the person to remove their own dentures by the caregiver reminding them			0.0458	0.6286
Caregiver reminds the person when they do not start to shave by themselves			0.0212	0.5936
Enable the person to take their medication at the correct time by a family member or helper handing over the medication each time it is required			−0.1312	0.5884
Point to the supermarket information sign and tell the person what they can buy and where they can buy it			0.2008	0.5187
Ensure there is no toothpaste remaining on the person's face by instructing the person to check in the mirror			0.1385	0.4670
Sum of squares of factor structure after rotation			3.9936	3.2859
Contribution ratio			32.16%	8.83%
Cumulative contribution ratio			32.16%	40.99%

Three benefits were extracted relating to care for people with daily life disabilities in Alzheimer's type dementia and moderate cognitive impairment (Table 3). The first factor was named “Opportunities for completion of a task are provided with verbal communication: create opportunities to enable the person to complete the ADL by themselves,” the second factor was named “Marks: place marks on the area or aspect of necessities for daily living that you want the person to focus on,” and the third factor was named “Arrange the environment with verbal communication: speak to the person and arrange their living environment together.” Cronbach's *α* coefficient demonstrated internal consistencies for the three factors of 0.920, 0.824 and 0.749, respectively, while the cumulative contribution ratio was 53.69%.

**Table 3:** Benefits of care for daily living disabilities in people with Alzheimer's type dementia and moderate cognitive impairment

Variable			Factor 1	Factor 2	Factor 3
Factor 1: Opportunities for completion of a task is provided with verbal communication: create opportunities to enable the person to complete the activities of daily living by themselves					
Cronbach's *α*	0.9203				
After teeth brushing, instruct the person to rinse out their mouth with water, to ensure the person rinses out their mouth			0.7894	−0.0335	0.0222
When the person does not wash their hands, remind them by saying “let's wash our hands”			0.7703	−0.0846	−0.0087
When the person has only washed part of their face, remind them to wash their whole face			0.7684	−0.0521	0.0379
Remind the person to make sure they lather the soap to cover their whole hands			0.7665	−0.0079	0.0084
Remind the person to shave the other side of their face when they have only shaved one side			0.6600	0.1382	0.0208
The caregiver verbally communicates how to soak in the bathtub			0.6546	0.1599	−0.0255
When the person tries to wear their pants as a jacket tell them in words that “they are pants, so let's wear them on your legs”			0.6531	0.0866	−0.0097
When the person is unsure of where to wash, remind them of the areas they haven't washed			0.6094	0.1311	0.0381
When the person does not clean their dentures themselves, lead them to the bathroom and remind them to clean the dentures			0.5751	0.1717	0.0639
Ensure the person understands that hot drinks are hot by telling them in words			0.5714	0.1046	−0.0324
Inform the person that it is daytime while looking at the scenery outside and encourage them to change out of their night clothes			0.4769	0.2200	0.0377
Factor 2: Marks: place marks on the area or aspect of necessities for daily living that you want the person to focus on					
Cronbach's *α*	0.8240				
When the person does not know the position of the switch on the electric shaver, affix a mark to make it easier to see			0.0475	0.7504	0.0132
Enable the person to differentiate between shampoo and other containers by changing the colour of the containers, writing the names in large letters, etc.			0.0844	0.7064	−0.0074
Ensure the person does not forget to extinguish flames by affixing a note “be careful with fire” in a visible location			−0.0506	0.6604	0.0938
Enable the person to adjust the water temperature by labelling the faucet to make it easy to see			0.0462	0.6377	−0.0105
Indicate a landmark on the target location and instruct the person to aim for that landmark			0.1266	0.5276	−0.0567
Factor 3: Arrange the environment with verbal communication: speak to the person and arrange their living environment together					
Cronbach's *α*	0.749				
When the person does not notice their house is dirty, remind them and clean it together			−0.0398	−0.0062	1.0194
The caregiver reminds the person how to tidy up and tidies up together with the person			0.2355	0.0635	0.4716
Sum of squares of factor structure after rotation			7.1849	5.5455	3.1340
Contribution ratio			16.07%	32.37%	5.25%
Cumulative contribution ratio			16.07%	48.44%	53.69%

Three factors on usefulness were extracted relating to care for people with daily life disabilities in Alzheimer's type dementia and severe cognitive impairment (Table 4). The first factor was named “Explain the process in the order of each individual action: explain the order of actions that comprise one activity of daily life and specifically show those actions,” the second factor was named “Prevent non‐starts and interruptions: devise techniques in advance to ensure activities of daily life are not non‐started or interrupted,” and the third factor was named “Confirm intention: slowly confirm the intentions of the person.” Cronbach's *α* coefficient demonstrated internal consistencies for the three factors of 0.948, 0.908 and 0.862, respectively, while the cumulative contribution ratio was 45.57%.

**Table 4:** Benefits of care for daily living disabilities in people with Alzheimer's type dementia and mild cognitive impairment

Variable			Factor 1	Factor 2	Factor 3
Factor 1: Explain the process in the order of each individual action: explain the order of actions that comprise one activity of daily life and specifically show those actions					
Cronbach's *α*	0.9480				
Place marks to remind the person of the order to place their hands and feet when getting into a car			0.8051	−0.1285	−0.0535
Display containers in order to ensure the person applies their makeup in the correct order			0.7968	−0.1131	0.0415
Get the person to look at the labels on clothing to ensure they understand left, right, back and front			0.7810	−0.0524	−0.0694
Explain a series of transfer movements and ensure the person understands the movements			0.7615	−0.1475	0.1082
If a person's posture is crooked and they make no attempt to correct it themselves, verbally remind them to correct their posture			0.7305	−0.0420	0.0080
Inform the person of the seated position with words and gestures, to ensure they sit back in their seat			0.6773	0.0060	0.0451
Ask the caregiver to observe the person brushing their teeth to ensure they understand the correct teeth brushing method			0.6681	0.0147	0.0776
When the person wears their shoes on the wrong feet, place marks on the shoes to ensure the person can tell left from right			0.6175	0.1223	−0.0563
Use words and gestures to ensure the person does not use shampoo to wash their face			0.6099	0.0950	−0.0034
When the person does not open their mouth to insert their dentures, remind them to ensure they are aware that they are putting in dentures			0.5974	0.0433	0.1497
When the person refuses to take their medication, inform them that you want them to take the medication as per the instructions from their attending physician			0.5811	−0.0121	0.0345
When the person wears their underwear on top of their clothes remind them after bathing, so they can wear their clothes appropriately			0.5454	0.0716	0.0154
When the person is unaware that it is a toothbrush, the caregiver stands beside the person and asks them to watch them brushing their teeth			0.5307	0.1951	0.0527
Ensure the person sits in an appropriate position on the toilet bowl by affixing plastic tape in the standing position			0.5278	0.2096	−0.1810
When the person does not know the way to open or close makeup lids, the caregiver communicates this information with words and gestures			0.4983	0.1707	0.1206
Place marks in the toilet bowl for men to enable them to urinate aiming for the mark			0.4914	0.1509	−0.1099
Separate the process into single units and communicate the process with words and actions, to enable the person to understand the way to remove their clothes and the order of removal			0.4884	0.2247	0.0914
When the person does not understand what is arranged on the table, verbally communicate the menu			0.4826	0.1603	0.0711
When the person continues to shave even after completing shaving, remind them “you have already finished shaving”			0.4584	0.1980	0.0188
Show the person where to grasp the hand rails to enable them to sit in an appropriate position on the toilet			0.4040	0.2323	−0.0301
Factor 2: Prevent non‐starts and interruptions: devise techniques in advance to ensure activities of daily life are not non‐started or interrupted					
Cronbach's *α*	0.9080				
When the person tries to eat by grasping with their hands or placing their mouth on the bowl, make it easier for them to eat by making it into a graspable shape			−0.0376	0.7385	0.0193
When the person is distracted by the pattern on a plate and cannot concentrate on their meal, change to a plate without a pattern			−0.0251	0.7345	−0.1035
When the person cannot put an appropriate amount of food in their mouth, adjust the size of the spoon or the size of the bowl			−0.0389	0.6859	0.1091
When the person needs time to open their mouth, get the person to put food into their mouth to match the timing of mouth opening			−0.0017	0.6766	0.0807
Interpret the signs for bowel movement and take the person to the toilet			−0.0267	0.6698	0.0360
Interpret the signs of a desire to urinate and take the person to the toilet			−0.1061	0.6433	0.1092
When the person does not start eating, or does not attempt to open their mouth, assist them with the first mouthful to start them eating			0.1474	0.6133	−0.0247
When the person does not put their hands under the faucet, take one hand slowly and wet it with lukewarm water			0.1680	0.6066	−0.0588
When the person is unable to cut or divide their food into small pieces, cut their food after asking “shall I cut it into small pieces?”			0.1377	0.5933	0.0019
When the person does not attempt to pick up their chopsticks or spoon, the caregiver should give the utensils to the person			0.1551	0.5677	0.0148
When the person does not understand to bring their hands to their face, wipe their face with a damp towel			0.0042	0.5249	0.0162
When the person is surprised by the water pressure of the shower, put hot water in a basin and use that instead			0.0965	0.4579	0.0743
When the person does not know where to hold a cup, use a cup with a handle			0.2160	0.4279	0.0712
Factor 3: Confirm intention: slowly confirm the intentions of the person					
Cronbach's *α*	0.8620				
Wait patiently until the person is able to express their intention themselves			0.0380	−0.0439	0.8060
Guess the person's feelings and check by asking them if they are feeling that way			−0.0039	0.0786	0.7588
Speak to the person in a rhythm that makes it easier for them to speak			−0.0527	0.1551	0.7257
When the person is unable to answer a question, ask again using easy to understand expressions			0.0651	0.1213	0.6228
Sum of squares of factor structure after rotation			11.3071	9.9958	7.2215
Contribution ratio			35.90%	7.09%	3.30%
Cumulative contribution ratio			35.90%	42.99%	45.57%

## 4 DISCUSSION

Various types of dementia, including Alzheimer's type dementia, first manifest as generalized attention disorder, characterized by the inability to pay attention to necessary tasks, maintain attention and appropriately select and distribute attention (Japan Society of Neurology, 2017). Therefore, support for generalized attention disorder is beneficial care for daily life disabilities. The first factor could be described as care that makes it easier for a person with Alzheimer's type dementia to turn their attention to matters by arranging items required for daily life in a simplified format. Therefore, the first factor is support for generalized attention disorder in people with Alzheimer's type dementia.

Maintaining, selecting and distributing attention are necessary conditions for establishing execution function, which is essential for execution of daily tasks, including ADL (Nakaaki & Sato, 2015). This means that once generalized attention disorder is supported with the first factor as the foundation, the second factor then supports the execution function disorder to progress through a series of steps. Execution function is formed from the four stages of decision‐making: starting an action, planning an action, executing an action and making adjustments if an unexpected situation occurs (Lezak et al., 2004; Walsh, 1991). Execution function disorder manifests from the first stages of Alzheimer's type dementia in the same way as generalized attention disorder, but in people with Alzheimer's type dementia with mild cognitive impairment, phased support of instantaneous planning and execution is particularly beneficial for implementing individual daily tasks in ADL.

Furthermore, with the progression of generalized attention disorder and execution function disorder, it becomes increasingly difficult to have intention for each daily task and to plan and execute those tasks. Therefore, care that encourages verbal communication of actions required for daily tasks to a person with Alzheimer's type dementia who is still able to understand verbal communication is beneficial as care for people with daily life disabilities in Alzheimer's type dementia. This is listed in the first factor: “Opportunities for completion of a task are provided with verbal communication: create opportunities to enable the person to complete the ADL by themselves.”

However, language disorders manifest from the early stage of Alzheimer's type dementia (Japan Society of Neurology, 2017) and symptoms of the ageing process and diseases also tend to appear in the vision and hearing of older people. Therefore, use of verbal communication only may be inadequate when providing care to people with Alzheimer's type dementia with moderate cognitive impairment. Thus, attaching marks to necessities of daily living that are visually easy to understand and notice, as listed in the second factor, is considered to be a beneficial method of care. These methods have been devised to make people with dementia more easily aware of their environment. It is important for people with dementia, including Alzheimer's type dementia, to live in a supportive and therapeutic environment (Calkins, 2018; Chau et al., 2018). The World Health Organization′s (2002) International Classification of Functioning states that mutual interaction between humans and their environment is important and it presents the importance of social models to improve the environment to overcome disorders that reduce life functions. This second factor, “Marks: place marks on the area or aspect of necessities for daily living that you want the person to focus on,” is a method to improve the environment in line with the level of cognitive impairment of the person with Alzheimer's type dementia, so it can be considered as care based on the ICF social model.

The third factor, “Arrange the environment with verbal communication: speak to the person and arrange their living environment together,” can similarly be considered as a method to improve the environment in line with the level of cognitive impairment of the person with Alzheimer's type dementia based on the ICF social model. The caregiver cleaning and arranging the living environment in the house together with the person with Alzheimer's type dementia cognitive impairment is essential to maintain a healthy living environment. It has also been reported in Japan that people with dementia with high ADL and low cognitive function and people with dementia who have little communication with surrounding caregivers and their peers in the same facility are prone to develop BPSD and those with BPSD have a high incidence of agitation/aggression (Arai, Ozaki, & Katsumata, 2017). People with Alzheimer's type dementia with moderate cognitive impairment are prone to developing imbalanced ADL and cognitive function and this is the stage where BPSD tend to occur, so the third factor is considered effective from the perspective of preventing BPSD.

“Explain the process in the order of each individual action: explain the order of actions that comprise one activity of daily life and specifically show those actions” was extracted as the first factor for care of people with daily life disabilities in Alzheimer's type dementia and severe cognitive impairment and it includes care that mainly uses the verbal and written word or devises methods to be able to appropriately use daily necessities. These results are generally consistent with the six‐stage assessment adopted by Sato et al. (2011), which is a method for assessing self‐care disorders in dementia patients that reflect executive function disorders, assessing each self‐care item, including dressing, bathing, eating and toileting based on the following six‐stage assessment: Independent, Requires encouragement to start, Requires induction with appropriate instructions, Requires induction with instructions for each stage, Requires induction with instructions for each stage and appropriate assistance for actions, Requires appropriate assistance for actions at each stage. However, these factors do not include care that provides appropriate assistance for actions at each stage, as advanced care practitioners for dementia prioritize the importance of people with Alzheimer's type dementia implementing actions themselves and, moreover, have been successful in implementing such behaviour.

However, a person with Alzheimer's type dementia with severe cognitive impairment not only has more severe execution function disorder, but they also develop agnosia and apraxia, which makes them unable to start daily tasks themselves or causes them to abandon ADL partway through. Even in these circumstances, support was provided to enable people with this level of disability to start and continue daily tasks themselves by making changes to daily necessities to support cognitive impairment, ascertaining the person's toileting rhythms and eating rhythms and enabling them to enjoy the experience of deliciousness and warmth when starting daily tasks, rather than simply assisting their action for each stage of the process. The self‐esteem of people with dementia tends to be adversely affected when ADL become difficult due to the effect of cognitive impairment (Dos Santos, Rocha, Fernandez, de Padua, & Reppold, 2018; Suwa, Otani, Tsujimura, Nogawa, & Shiya, 2018). However, care using not only the first factor, “Explain the process in the order of each individual action: explain the order of actions that comprise one activity of daily life and specifically show those actions,” but also the second factor, “Prevent non‐starts and interruptions: devise techniques in advance to ensure activities of daily life are not non‐started or interrupted,” enables the person to execute ADL, which may also maintain or improve their self‐esteem.

Furthermore, the third factor was “Confirm intention: slowly confirm the intentions of the person.” The first and second factors were simply care methods to support a person with Alzheimer's type dementia with severe cognitive impairment to execute daily tasks themselves. However, in reality, as the cognitive impairment progresses, including execution function disorders and language disorders, many people with dementia become unable to smoothly indicate their intention to perform various daily tasks. It is easy to focus on the ability to consent to medical treatment, where people with dementia are assessed during medical treatment and care using assessment tools such as the Macarthur Competence Assessment Tool for Treatment (Grisso & Appelbaum, 1998) and Competency to Consent to Treatment Instrument (Marson, Ingram, Cody, & Harrell, 1995). However, when providing support to a person with Alzheimer's type dementia in these situations in a way that enables the person to retain their dignity, it is possible to check the intention of the person with dementia and obtain their consent regarding matters. This includes confirming that it is acceptable to start each daily task, continue the daily task, complete the daily task and use convenient daily necessities to execute daily tasks. These actions respect the intention of people with dementia even when they require assistance for all their daily tasks. The results of this study suggest that these practices are beneficial for maintaining the autonomy of a person with Alzheimer's type dementia when performing daily tasks.

The reliability of the above factors as characteristics of care methods that respect the autonomy and independence of people with daily life disabilities is shown by Cronbach's *α* coefficient demonstrating internal consistency. The Cronbach's *α* coefficient for almost all the factors was 0.8 or higher, so it can be said that these factors demonstrate the characteristics of the care methods. However, Cronbach's *α* coefficient was 0.774 for the second factor, “Communication using verbal language on what should be done next: speak to the person and inform them of what they should do next,” for care of people with daily life disabilities in Alzheimer's type dementia and mild cognitive impairment. The Cronbach's *α* coefficient was also 0.749 for the third factor, “Arrange the environment with verbal communication: speak to the person and arrange their living environment together,” for care of people with daily life disabilities in Alzheimer's type dementia and moderate cognitive impairment. These two factors are both methods of care that use verbal language to communicate with the person with Alzheimer's type dementia. Alsawy, Mansell, McEvoy, and Tai (2017) indicate that communication difficulties affect ADL and the personal relationship between the person and caregiver. Their report also stresses the importance of the relationship between the person with Alzheimer's type dementia and the caregiver and that the person feels respect from the caregiver during periods of communication. Factors that comprise verbal communication include the words that are used, length of each sentence, tone, tone of voice, timing of talking to someone and speed of the speech; therefore, how a person with Alzheimer's type dementia feels when spoken to by the caregiver and whether they will behave in accordance with what was said, is thought to change depending on the person's relationship with the caregiver. This is thought to be the reason that Cronbach's *α* coefficient only reached the level of 0.7 for the two factors that are care methods using verbal communication. However, Cronbach's *α* coefficient was 0.920 for communicating opportunities only via verbal language, the factor “Opportunities for completion of a task are provided with verbal communication: create opportunities to enable the person to complete the ADL by themselves.” Thus, it is necessary to clarify the specifics of appropriate verbal and non‐verbal communication for care of daily life disabilities.

### 4.1 Research limitations and issues

This study used data obtained from advanced care practitioners for dementia on support for multiple people with daily life disabilities in Alzheimer's type dementia and their envisioned care, so it may not accurately reflect actual care used for daily life disabilities. Also, this study did not consider the relationship of various factors including the detailed cognitive impairment condition of a person with Alzheimer's type dementia, treatment status with anti‐dementia drugs and onset status of BPSD.

It is essential to conduct a longitudinal study into people with daily life disabilities in Alzheimer's type dementia and the associated care, to undertake further investigation into the benefits of care from the perspective of specific daily life disabilities and care, to determine how to maintain or improve autonomy and independence and to ascertain whether such improvements are even possible. It is also important to assess self‐care in ADL based on cognitive impairment, including execution function disorder and to develop care strategies that respect autonomy and independence. This will enable a method to move away from simply classifying care for ADL for people with dementia in Japan into independent, partial care and total care.

## 5 CONCLUSION

This study clarified the characteristics of care methods that respect the autonomy and independence of people with daily life disabilities in Alzheimer's type dementia based on the severity of their condition implemented by advanced dementia care practitioners in Japan. The results of factor analysis extracted the characteristics of “Simplicity of necessities: integrate the necessities for daily living in a simplified format” and “Communication using verbal language on what should be done next: speak to the person and inform them of what they should do next” relating to care for people with daily life disabilities in Alzheimer's type dementia and mild cognitive impairment. “Opportunities for completion of a task are provided with verbal communication: create opportunities to enable the person to complete the ADL by themselves,” “Marks: place marks on the area or aspect of necessities for daily living that you want the person to focus on” and “Arrange the environment with verbal communication: speak to the person and arrange their living environment together” were clarified as characteristics relating to care for people with daily life disabilities in Alzheimer's type dementia and moderate cognitive impairment. “Explain the process in the order of each individual action: explain the order of actions that comprise one activity of daily life and specifically show those actions,” “Prevent non‐starts and interruptions: devise techniques in advance to ensure activities of daily life are not non‐started or interrupted” and “Confirm intention: slowly confirm the intentions of the person” were listed as characteristics of care for people with Alzheimer's type dementia with severe cognitive impairment. Based on the aforementioned characteristics, it is important to courteously support daily tasks, including eating meals, bathing and toileting, to enable people with Alzheimer's type dementia to retain their dignity.

## CONFLICT OF INTEREST

No conflict of interest has been declared by the authors.

## AUTHOR CONTRIBUTIONS

SS, AY, MU, TY, YH, MS: Substantial contributions to conception and design, or acquisition of data, or analysis and interpretation of data; drafting the manuscript or revising it critically for important intellectual content; final approval of the version to be published. Each author should have participated sufficiently in the work to take public responsibility for appropriate portions of the content and agreed to be accountable for all aspects of the work in ensuring that questions related to the accuracy or integrity of any part of the work are appropriately investigated and resolved.
